# Effect of feeding regimens on circadian rhythms: Implications for aging and longevity

**DOI:** 10.18632/aging.100116

**Published:** 2010-01-11

**Authors:** Oren Froy, Ruth Miskin

**Affiliations:** ^1^ Institute of Biochemistry, Food Science and Nutrition, Robert H. Smith Faculty of Agriculture, Food and Environment, The Hebrew University of Jerusalem, Rehovot 76100, Israel; ^2^ Department of Biological Chemistry, Weizmann Institute of Science, Rehovot 76100, Israel

**Keywords:** clock, circadian rhythms, caloric restriction, intermittent fasting, metabolism, αMUPA, life span, aging

## Abstract

Increased longevity and improved health can be achieved in mammals by two feeding regimens, caloric restriction (CR), which limits the amount of daily calorie intake, and intermittent fasting (IF), which allows the food to be availablead libitum every other day. The precise mechanisms mediating these beneficial effects are still unresolved. Resetting the circadian clock is another intervention that can lead to increased life span and well being, while clock disruption is associated with aging and morbidity. Currently, a large body of evidence links circadian rhythms with metabolism and feeding regimens. In particular, CR, and possibly also IF, can entrain the master clock located in the suprachiasmatic nuclei (SCN) of the brain hypothalamus. These findings raise the hypothesis that the beneficial effects exerted by these feeding regimens could be mediated, at least in part, through resetting of the circadian clock, thus leading to synchrony in metabolism and physiology. This hypothesis is reinforced by a transgenic mouse model showing spontaneously reduced eating alongside robust circadian rhythms and increased life span. This review will summarize recent findings concerning the relationships between feeding regimens, circadian rhythms, and metabolism with implications for ageing attenuation and life span extension.

## I. Circadian rhythms, well-being, and life span

Organisms on earth evolved to restrict their activity to the night or day, being nocturnal or diurnal, respectively. By developing an endogenous circadian (*circa* - about; *dies* - day) clock, which can be entrained to external stimuli, primarily light, animals and plants ensure that physiological processes are performed at the appropriate, optimal time of day or night [1]. Adaptation to external conditions through clock entrainment imparts a survival advantage, as the organism can predict environmental changes [1-3]. The clock core machinery is self sustained, so that in the absence of external cues, e.g., in constant darkness, the endogenous rhythms free-run, generating cycles of approximately but not exactly 24 hours.

In mammals, the circadian clock influences nearly all aspects of physiology and behavior, such as sleep-wake cycles, cardiovascular activity, endocrine system, body temperature, renal activity, physiology of the gastrointestinal tract, and hepatic metabolism [1,2]. Epidemiological studies indicate that myocardial infarction, pulmonary edema, hypertensive crises, and asthma and allergic rhinitis attacks, all peak at certain times during the day [4-6]. Disruption of circadian coordination in humans or animals is manifested by hormone imbalance, some aspects of disease, and reduced life span [2,7-12].For instance, psychological and sleep disorders [2] and cardiovascular diseases [13,14] can be associated with irregular or dysfunctional circadian clock. Disruption of circadian coordination can also accelerate cancer proneness and malignant growth in animals and humans, suggesting that the circadian clock controls tumor progression [8-10]. In addition, symptoms seen in jet lagged travelers, e.g., fatigue, disorientation, and insomnia, or in shift workers, e.g., altered hormone profiles and morbidity, result from the constant need to extend wakefulness or to repeatedly invert the normal sleep-wake cycle [10,15,16]. Also, chronic reversal of the external light-dark cycle at weekly intervals resulted in a significant decrease in the survival time of cardiomyopathic hamsters [7]. Importantly, circadian rhythms change with normal aging in animals and humans, including a shift in the phase and decrease in amplitude [15-18]. By using a more direct approach, it was shown thatlongevity was diminished in golden hamsters carrying a 20 h-period mutation, *tau*, raised in 24 h light-dark cycles [19]. On the contrary, life span was extended in aged animals given fetal suprachiasmatic implants that restore higher amplitude rhythms [19-21]. Altogether, it seems that circadian disruption is associated with multiple negative manifestations, whereas resetting of circadian rhythms could lead to increased longevity. These findings, although largely correlative, point to a critical role for the circadian clock in maintaining normal peripheral physiology.

## II. The circadian clock

**Figure 1.**
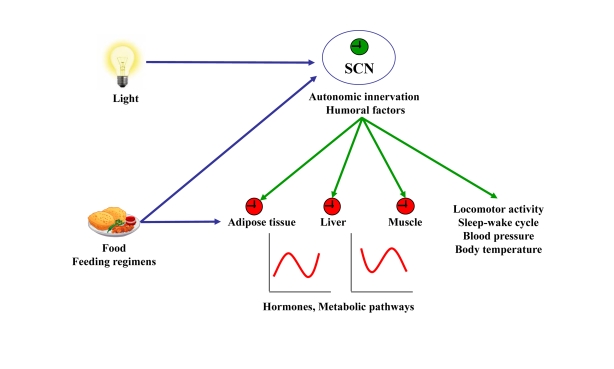
Resetting signals of the central and peripheral clocks. The SCN resets peripheral oscillators *via* humoral factors or autonomic innervation leading to circadian hormone expression and secretion and rhythmic activity of metabolic pathways. In addition, the SCN dictates rhythms of locomotor activity, sleep-wake cycle, blood pressure, and body temperature. Light, food, and feeding regimens affect either the central clock in the SCN or peripheral clocks. Input to central or peripheral clocks are in blue. Outputs from the central clock to the periphery are in green.

***A. The location of the mammalian circadian clock.*** In mammals, the central circadian clock is located in the suprachiasmatic nuclei (SCN), a distinct bilateral group of cells located in the anterior hypothalamus in the brain. Similar clock oscillators have been found in many peripheral tissues, such as the liver, intestine, heart, adipose tissue, retina and in various regions of the brain [2,22-24]. The SCN clock is composed of multiple, intracellular circadian oscillators, which, when synchronized, generate coordinated circadian outputs that regulate overt rhythms [25-28]. SCN oscillation is not exactly 24 h and it is necessaryto entrain the circadian pacemaker each day to the external light-dark cycle to prevent drifting (or free-running) out of phase. Light perceived primarily by melanopsin-expressing retinal ganglion cells transmit signals to the SCN *via* the retinohypothalamic tract (RHT) [2,29,30]. As a result, vasoactive intestinal polypeptide (VIP), an intrinsic SCN factor, acutely activates and synchronizes SCN neurons [31,32]. Synchronization among SCN neurons leads to the sending of signals to peripheral oscillators to prevent the dampening of circadian rhythms in these tissues. The SCN accomplishes this task *via* neuronal connections or circulating humoral factors [33] although the mechanisms are not fully understood (Figure 1). Several humoral factors expressed cyclically by the SCN, such as transforming growth factor α (TGFα) [34], prokineticin 2 (PK2) [35], and cardiotrophin-like cytokine (CLC) [36], have been shown to affect peripheral clocks. Their intracerebroventricular injection inhibits nocturnal locomotor activity, an SCN output. Complete electrical destruction of SCN neurons abolishes overall circadian rhythmicity in SCN-controlled tissues, because of the loss of synchrony among individual cells in the periphery and damping of the rhythm at the population level [37]. However, at the cellular level each cell oscillates, but with a different phase [37,38]. The fraction of cyclically expressed transcripts in each peripheral tissue ranges between 5-20% of the total population and the vast majority of these genes are tissue-specific [24,39-47]. These findings emphasize the circadian control over a large portion of the transcriptomes in peripheral tissues. Considering the circadian gene expression in peripheral tissues, it is difficult to determine whether the SCN clock drives these rhythmic patterns directly or indirectly by driving rhythmic feeding, activity, and/or body temperature, which, in turn, contribute to rhythms in gene expression in the periphery. It has been shown that for a peripheral tissue, such as the liver, signals both from the SCN clock or the local endogenous clock may control rhythmic gene expression [48,49].

***B. The biological clock at the molecular level.*** Genetic analysis of mutations affecting the clock in *Neurospora*, *Drosophila*, Cyanobacteria, *Arabidopsis*, and, recently, the mouse have paved the way for the identification of clock genes. In mammals, the clock is an intracellular mechanism sharing the same molecular components in SCN neurons and peripheral cells [3]. Generation of circadian rhythms is dependent on the concerted co-expression of specific clock genes. Transcriptional-translational feedback loops lie at the very heart of the core clock mechanism. Many clock gene products function as transcription factors, which possess PAS (PER, ARNT, SIM) and basic helix-loop-helix (bHLH) domains involved in protein-protein and protein-DNA interactions, respectively. These factors ultimately activate or repress their own expression and, thus, constitute self-sustained transcriptional feedback loops. Changes in concentration, subcellular localization, post-transcriptional microRNA regulation, posttranslational modifications (phosphorylation, acetylation, deacetylation, SUMOylation), and delays between transcription and translation are crucial in order to achieve a 24-h cycle [1,2,50-52].

**Figure 2.**
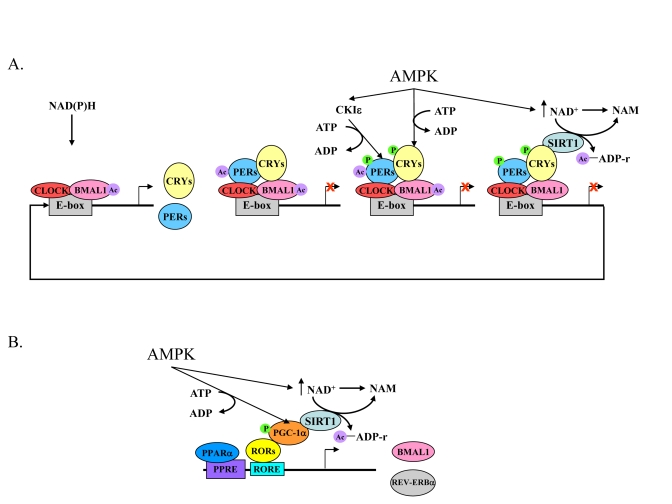
The core mechanism of the mammalian circadian clock and its link to energy metabolism. (**A**) High NAD(P)H levels promote CLOCK:BMAL1 binding to E-box sequences leading to the acetylation of BMAL1 and expression of Pers, Crys, and other clock-controlled genes. The negative feedback loop, PERs:CRYs, binds to CLOCK:BMAL1 and consequently PERs are acetylated. Activated AMPK leads to a rise in NAD+ levels, phosphorylation of CRYs, and phosphorylation of CKI?, which then phosphorylates the PERs. As a result of increased NAD+ levels, SIRT1 deacetylates PERs and BMAL1. This and the destabilization of phosphorylated PERs and CRYs relieves PERs:CRYs repression and another cycle starts. (**B**) Expression of Bmal1 and Rev-erbα genes are controlled by PPARα and binding of RORs to RORE sequences. RORs need a co-activator, PGC-1α, which is phosphorylated by activated AMPK. In parallel, AMPK activation leads to an increase in NAD+ levels, which, in turn activate SIRT1. SIRT1 activation leads to PGC-1α deacetylation and activation. Acetyl adenosine diphosphate ribose (Ac-ADP-r) and nicotinamide (NAM) are released after deacetylation by SIRT1.

In the mouse, the first clock gene identified, encodes the transcription factor CLOCK (Circadian Locomotor Output Cycles Kaput) [53], which dimerizes with BMAL1 (brain and muscle ARNT-like protein 1) to activate transcription. CLOCK and BMAL1, two bHLH-PAS transcription factors, are capable of activating transcription upon binding to E-box (5'- CACGTG -3') and E-box-like promoter sequences [2]. BMAL1 can also dimerize with other CLOCK homologs, such as neuronal PAS domain protein 2 (NPAS2), to activate transcription and sustain rhythmicity [54,55]. Amongst the regulatory targets of CLOCK:BMAL1 are the three *Period* genes (*Per*1, *Per*2, and *Per*3), which encode PAS domain factors, and two *Cryptochrome* genes (*Cry1* and *Cry2*). PERs and CRYs function as negative regulators, blocking CLOCK:BMAL1-mediated transcriptional activation [2,56] (Figure 2A).Thus, CLOCK:BMAL1 heterodimers bind to E-box sequences and mediate transcription of a large number of genes including those of the negative feedback loop *Per*s and *Cry*s. When PERs and CRYs are produced in the cytoplasm, they oligomerize after reaching an appropriate concentrationand translocate to the nucleus to inhibit CLOCK:BMAL1-mediated transcription. All the aforementioned clock genes exhibit a 24-h oscillation in SCN cells and peripheral tissues, except for *Clock* that has been shown not to oscillate in the SCN [50]. Recent studies have demonstrated that CLOCK has intrinsic histone acetyltransferase activity, suggesting that rhythmic activation of chromatin remodeling may underlie the clock transcriptional network [57,58]. Indeed, cyclic histone acetylation and methylation have been observed on the promoters of several clock genes [58-63]. In addition, CLOCK also acetylates several proteins of the core clock apparatus, thus, enabling cycles of acetylation and deacetylation, the latter activity involving SIRT1 will be discussed below (Figure 2).

Several other players appear to be important to sustain clock function. Casein kinase I epsilon (CKIε) phosphorylates the PER proteins and, thereby, enhances their instability and degradation [50,64-66]. CKIε also phosphorylates and partially activates the transcription factor BMAL1 [67].* Bmal1* expression is nega�tively regulated by the transcription factor reverse erythroblastosis virus α (REV-ERBα) [68], which recruits histone deacetylase (HDAC) complexes [69]. *Bmal1* expression is positively regulated by retinoic acid receptor-related orphan receptor α (RORα) and RORγ [70]*via* the ROR response element (RORE) [71]. Thus, *Bmal1 *oscillation is driven by a rhythmic change in RORE occupancy by RORs and REV-ERBα. This alternating occupancy occurs due to the robust rhythmic levels of REV-ERBα, a result of direct transcriptional activation of the *Rev-erb*α gene by the heterodimer CLOCK:BMAL1 [68]. Indeed, mice deficient in RORα or REV-ERBα have impaired circadian rhythms of locomotor activity and clock gene expression [68,70] (Figure 2B).

## III. The biological clock and energy homeostasis

## *A. Circadian rhythms and metabolism*

 The circadian clock has been reported to regulate metabolism and energy homeostasis in peripheral tissues [72,73]. This is achieved by mediating the expression and/or activity of certain metabolic enzymes and transport systems [74,75] involved in metabolic pathways [76-80]. In addition, a large number of nuclear receptors involved in lipid and glucose metabolism has been found to exhibit circadian expression [81]. Many hormones involved in metabolism, such as insulin [76], glucagon [82], adiponectin [83], corticosterone [84], leptin, and ghrelin [85,86], have been shown to exhibit circadian oscillation. Leptin, an adipocyte-derived circulating hormone, acts at specific receptors in the hypothalamus to suppress appetite and increase catabolism. Leptin exhibits striking circadian patterns in both gene expression and protein secretion, with peaks during the sleep phase in humans [87]. Neither feeding time nor adrenalectomy affected the rhythmicity of leptin release. However, ablation of the SCN has been shown to eliminate leptin circadian rhythmicity in rodents, suggesting that the central circadian clock regulates leptin expression [88]. Receptors for leptin and ghrelin are present on SCN cells [89-91], so it is possible that these hormones bind directly to SCN neurons, similarly to their effect on the orexigenic neuropeptide Y (NPY) and agouti-related protein (AgRP) neurons. Indeed, exogenous leptin was reported to phase-advance rhythms of neuronal firing in rat SCN slices [92].Activation of ventromedial arcuate nucleus (vmARC) neurons by systemic administration of the ghrelin mimetic growth hormone-releasing peptide 6 combined with SCN tracing showed that vmARC neurons transmit feeding-related signals to the SCN [90]. This injection induced Fos in the vmARC and resulted in attenuation of light-induced phase delay in mice and light-induced Fos expression in the SCN in rats [93]. Administration of ghrelin *in vitro* to SCN slices or SCN explants from *Per2::luc *transgenic mice caused phase shifts in *Per2::luc *reporter gene expression. However, intra-peritoneal injection of ghrelin to wild type mice caused phase shifts only after 30 h of food deprivation, but not when the mice were fed *ad libitum *[94]. Thus, it seems that ghrelin and leptin may affect the SCN directly or through their effect on the ARC, which is then relayed to the SCN.

Experiments using cultured cells have suggested that the cellular redox state is capable of influencing rhythms [95]. CLOCKand itshomolog NPAS2 can bind efficiently to BMAL1 andconsequently to E-box sequences in the presence of reduced nicotinamide adenine dinucleotides (NADH and NADPH) (Figure 2A). On the other hand, the oxidized forms of the nicotinamide adenine dinucleotides (NAD^+^ and NADP^+^) inhibit DNA binding of CLOCK:BMAL1 or NPAS2:BMAL1 [95,96]. As the NAD(P)^+^/NAD(P)Hredox equilibrium depends on the metabolicstate of the cell, this ratio could dictate the binding of CLOCK/NPAS2:BMAL1 to E-boxes and result in phase-shifting of cyclic gene expression [74,95,96]. NAD^+^ is also required for the activation of SIRT1, a deacetylase involved in clock modulation, as will be discussed below.

## *B. The circadian clock and crucial metabolic factors are tightly linked*

BMAL1 : Circadian clocks have been shown to be present in several fat tissues, including inguinal white adipose tissue, epididymal white adipose tissue, and brown adipose tissue [45,97,98]. Recent transcriptome studies revealed rhythmic expression of clock and adipokine genes, such as resistin, adiponectin, and visfatin, in visceral fat tissue [83]. Recent molecular studies established the involvement of BMAL1 activity in the control of adipogenesis and lipid metabolism in mature adipocytes. Embryonic fibroblasts from* Bmal1*^-/- ^knockout mice failed to differentiate into adipocytes. Loss of BMAL1 expression led to a significant decrease in adipogenesis and gene expression of several key adipogenic/lipogenic factors. Furthermore, over-expression of BMAL1 in adipocytes increased lipid synthesis activity. Thus, BMAL1, a master regulator of circadian rhythms, plays important roles in the regulation of adipose differentiation and lipogenesis in mature adipocytes [99].

REV-ERBα : Another important candidate to link the circadian clock with lipid metabolism is REV-ERBα. This pro-adipogenic transcription factor, whose levels increase dramatically during adipocyte differentiation [100], exhibits striking diurnal variations in expression in murine adipose tissue [101] and rat liver [102]. During adipocyte differentiation, REV-ERBα acts downstream of the differentiation factor peroxisome proliferator receptor-γ (PPARγ) by facilitating gene expression of PPARγ target genes [103,104]. Ectopic REV-ERBα expression in 3T3L1 pre-adipocytes promotes their differentiation into mature adipocytes [103]. In addition to its role in lipid metabolism and adipocyte differentiation, REV-ERBα is a component of the core clock apparatus, as mentioned above (Figure 2B). It acts as a negative regulator of *Bmal1* expression, and its encoding gene, *Rev-erb*α, is directly activated by the CLOCK:BMAL1 heterodimer [68].

PPARα : Peroxisome proliferator-activated receptor α (PPARα) is a member of the nuclear receptor family that plays a unique role at the intersection of circadian and lipid metabolic pathways. The CLOCK:BMAL heterodimer mediates the expression of PPARα, which subsequently binds to the peroxisome-proliferator response element (PPRE) and activates transcription of *Bmal1 *[105-107] (Figure 2B). PPARα also regulates transcription of genes involved in lipid and glucose metabolism upon binding of endogenous free fatty acids [108,109]. Thus the circadian rhythmicity of PPARα provides an example of a reciprocal link between circadian and lipid metabolic processes.

PPARγ coactivator (PGC-1α) : PGC-1α, a transcriptional co-activator that regulates energy metabolism, is rhythmically expressed in the liver and skeletal muscle of mice. PGC-1α stimulates the expression of *Bmal1* and *Rev-erbα*, through co-activation of the ROR family of orphan nuclear receptors [110,111] (Figure 2B). Mice lacking PGC-1α show abnormal diurnal rhythms of activity, body temperature, and metabolic rate, due to aberrant expression of clock genes and those involved in energy metabolism. Analyses of PGC-1α-deficient fibroblasts and mice with liver-specific knockdown of PGC-1α indicate that it is required for cell-autonomous clock function [110].

AMP-activated protein kinase (AMPK) : AMPK could be another important link that integrates the circadian clock with metabolism. AMPK is a sensor of the energy status within cells, which upon activation acts to restore energy balance [112,113]. This is done in part by modulating NAD^+^ levels and SIRT1 activity [114,115]. AMPK has been found to directly phosphorylate Ser-389 of CKIε in Rat-1 fibroblasts, resulting in increased CKIε activity and degradation of mPER2 (Figure 2A). mPER2 degradation led to a phase advance in the circadian expression pattern of clock genes [116]. AMPK has also been shown to phosphorylate and destabilize mCRY1 in mouse fibroblasts, leading to altered circadian rhythms [117] (Figure 2A). In addition, the expression profile of clock-related genes, such as *Per1* and *Cry2* in skeletal muscle, as well as the diurnal shift in energy utilization, is impaired in AMPKγ_3_ subunit knockout mice in response to 5-amino-4-imidazole-carboxamide riboside (AICAR), an AMPK activator [118]. In addition to its intracellular role, AMPK is involved in whole body energy metabolism by regulating the response to feeding in the hypothalamus [112]. In this brain area, AMPK activation is inhibited by leptin and insulin, hormones which suppress feeding, whereas it is activated under starvation by ghrelin, a hormone primarily produced by the stomach that leads to increased food intake [119-122].

SIRT1 : Another protein recently found to link metabolism with the circadian clock is SIRT1. This is the mammalian ortholog of yeast Sir2, an NAD^+^-dependent histone deacetylase involved in transcriptional silencing and genome stability in yeast [123,124]. Sir2 or its ortholog enzymes are involved in life span extension and the response to caloric restriction in yeast, *Caenorhabditis elegans*, *Drosophila* [123,125], and mice [115,126]. The dependence on NAD^+^ as a cofactor for catalysis is thought to link SIRT1 activity to the energy state of the cell [127]. Non-histone substrates of SIRT1, as found in C2C12 myotubes, include regulatory molecules that modulate energy metabolism, such as PPARγ and PGC-1α [114], key factors that regulate the core molecular clock (Figure 2). Recent studies showthat SIRT1 interacts directly with CLOCK and deacetylates BMAL1 and PER2 in cultured fibroblasts [128,129] (Figure 2A). It seems that after binding to E-box, CLOCK and CBP/p300 acetylate histones H3 and H4 [57] and BMAL1 [130]. BMAL1 acetylation potentiates its binding by the repressive PER/CRY complex [130] and, as a result, PER2 is acetylated [128]. When acetylated, PER2 [128] and possibly BMAL1 [129] are more stable. SIRT1 then becomes activated and deacetylates BMAL1, PER2, and histones [131]. Deacetylated PER2 is further phosphorylated and degraded and a new cycle begins (Figure 2A). It has also been shown that the CLOCK:BMAL1 heterodimer regulates the circadian expression of NAMPT (nicotinamide phosphoribosyl-transferase), a rate-limiting enzyme in the NAD^+^ salvage pathway. SIRT1 is recruited to the *Nampt* promoter and contributes to the circadian synthesis of its own coenzyme [132]. Most recently, it has been shown that AMPK enhances SIRT1 activity by increasing cellular NAD^+^ levels, resulting in the deacetylation and modulation of the activity of downstream SIRT1 targets [114]. Thus, the levels of NAD^+^ together with the cycling of SIRT1 can determine the activity and robustness of clock gene transcription at least in cultured cells.

## *C. Clock mutations and metabolic disorders*

The most compelling linkage between metabolic disorders and the circadian clock is demonstrated by the phenotypes of clock gene mutants and knockouts. Homozygous C57BL/6J *Clock*^Δ^^19^ mice, with a truncated exon 18 and deleted exon 19 of the *Clock* gene, have a greatly attenuated diurnal feeding rhythm, are hyperphagicand obese, and develop a metabolic syndrome of hyperleptinemia,hyperlipidemia, hepatic steatosis, and hyperglycemia [133]. Loss of circadian rhythms in *Clock*^Δ^^19^ mutant mice was accompanied by attenuated expression of hypothalamic peptides associated with energy balance, such as ghrelin and orexin [133]. Insulin administration caused significantlygreater hypoglycemia in *Clock*^Δ^^19 ^mutant mice than in wildtype mice [134]. Increased insulin sensitivity was also seen in *Clock*^Δ19^ mutant mice of the BALB/c/CBA background that preserve rhythmicity in melatonin production [135]. In *Clock*^Δ^^19 ^on an Jcl:ICR background, serum levels of triglyceride and free fatty acids were significantly lower than in wild type control mice, whereas total cholesterol and glucose, insulin, and leptin levels did not differ [136]. Unlike C57BL/6J* Clock*^Δ^^19^ mutant mice, Jcl:ICR* Clock*^Δ^^19 ^mutantmice were not obese, had low or normal fasting plasmaglucose, low plasma free fatty acids, and normal plasma leptin. However, in Jcl:ICR* Clock*^Δ^^19 ^mutant mice, high fat diet amplified the diurnal variation in glucose tolerance and insulin sensitivity, and obesity was attenuated through impaired dietary fat absorption [136]. Although the effects on metabolism were variable due to strain differences, the overall picture is that disruption of the clock gene leads to disruption of metabolic pathways.

*Bmal1^-/-^* knockout mice, similarly to C57BL/6J* Clock*^Δ^^19^mutant mice, exhibited suppressed diurnal variations in glucose and triglycerides as well as abolished gluconeogenesis. Liver-specific deletion of *Bmal1* showed a direct effect of the liver clock on glucose metabolism, as exhibited by hypoglycemia during fasting, exaggerated glucose clearance, and loss of rhythmic expression of hepatic glucose regulatory genes [137]. Although recovery from insulin-induced hypoglycemia was impaired in C57BL/6J* Clock*^Δ^^19 ^mutant and *Bmal1^-/-^* knockout mice, the counter-regulatory responses of corticosterone and glucagon were retained [134].

Mutation in another central clock gene, *Per2* (*mPer2^-/-^* mice), exhibited no glucocorticoid rhythm even though the corticosterone response to hypoglycemia was intact. In addition, the diurnal feeding rhythm was absent in these mice. Although food consumption was similar during the light and dark periods on high fat diet, *mPer2^-/-^* mice developed significant obesity [138].

## IV. Effect of feeding regimens on circadian rhythms

In addition to light, feeding regimens have been reported to affect the clocks in the SCN and/or the periphery.

## A. Restricted feeding (RF)

**Figure 3.**
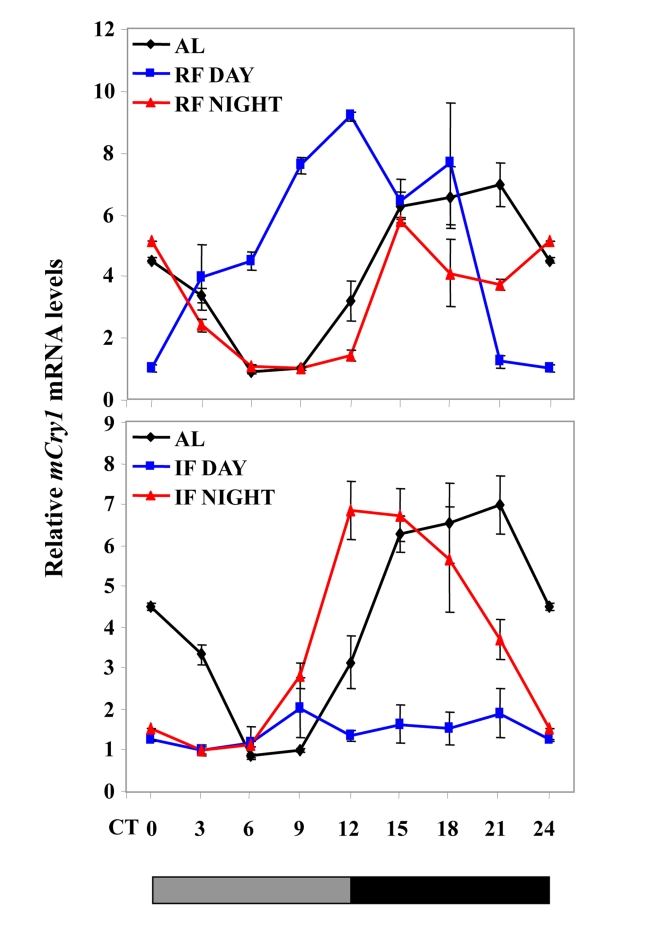
Effect of night vs. day RF and night vs. day IF on clock gene expression. Expression of a representative clock gene *mCry1* was measured in the liver of C57BL mice during *ad libitum* (AL), day and night RF, and day and night IF.Total RNA extracted from liver tissue collected every 3 h around the circadian cycle (mean ± SEM; n=3 for each time-point and each mouse group) was reverse transcribed and analyzed by quantitative real time PCR. Clock gene levels were normalized using *Gapdh* as the reference gene. The grey and black bars designate the subjective light and dark cycles, respectively.

RF limits the time and duration of food availability with no calorie reduction [3,74,139].Animals, which receive food *ad libitum* everyday at the same time for only a few hours, adjust to the feeding period within a few days [49] and can consume their daily food intake during that limited time [140,141]. Restricting food to a particular time of day has profound effects on the behavior and physiology of animals. Many physiological activities that are normally dictated by the master clock in the SCN are altered by RF, such as hepatic P450 activity, body temperature, locomotor activity, and heart rate [142-145]. 2-4 h before the meal, the animals display food anticipatory activity (FAA), which is typifiedby an increase in locomotor activity, body temperature, corticosterone secretion,gastrointestinal motility, and activity of digestive enzymes [140,146-148], all are known output systems of the biological clock. RF is dominant over the SCN and drives rhythms in arrhythmic and clock mutant mice and animals with lesioned SCN, regardless of the lighting conditions [142,143,148-151]. In most incidents, RF affects the core clock apparatus in peripheral tissues, such as liver (Figure 3), kidney, heart, and pancreas, with no effect on the central pacemaker in the SCN [3,74,139,143,150,152,153], causing uncoupling from the central pacemaker in the SCN. This suggests that nutritional regulation of clock oscillators in peripheral tissues may play a direct role in coordinating metabolic oscillations [154]. As soon as food availability returns to normal, the SCN clock, whosephase remains unaffected,resets the peripheral oscillators [152]. The location of this food-entrainable oscillator (FEO) has been elusive. Lesions in the dorsomedial hypothalamic nucleus (DMH) [155-158], the brain stem parabrachial nuclei (PBN) [156,159], and the core and shell regions of nucleus accumbens [160,161] revealed that these brain regions may be involved in FEO output, but they cannot fully account for the oscillation [162]. Neither vagal signals nor leptin are critical for the entrainment [163,164].CLOCK [165] or BMAL1 [166] and other clock genes [167] have been shown not to be necessary for food anticipatory activity. However, it has recently been demonstrated that *mPer2* mutant mice did not exhibit wheel-running food anticipation [168,169]. Recently, the FEO was suggested to be localized, in part, in ghrelin-secreting cells in the stomach [170]. Clearly, the localization and nature of the FEO and the effect of RF on circadian rhythms warrants further study.

The effect of RF on ageing and longevity has never been studied. Interestingly, the survival of Glasgow osteosarcoma-inoculated mice was prolonged under an RF regimen during the light period compared to those under the dark period or those fed *ad libitum* [171]. Also, RF modified the expression of genes involved in carcinogenesis and tumor progression, such as c-myc and p53 [172]. It remains to be determined whether RF feeding affects life span.

## *B. Calorie restriction (CR)*

CR, sometimes denoted dietary restriction (DR), refers to a dietary regimen low in calories without malnutrition, that restricts the daily amount of calories derived from carbohydrates, fats, or proteins usually to 60-75% of *ad libitum*-fed animals. CR extends the life span of diverse species, such as *C. elegans*, *Drososphila*, rodents [125,173,174], and recently monkeys [175]. CR in mice, rats, and monkeys prevents or delays the onset of major age-related diseases, such as cancer, diabetes, kidney disease, and cataracts [173,176]. In humans, long-termed CR results in sustained beneficial effects on major risk factors for atherosclerosis, type 2 diabetes, and inflammation [177]. The reduction of energy intake is considered to be the critical beneficial factor in the CR regimen [173]. Theories on how CR modulates aging and longevity abound, but the exact mechanism is still unclear [178]. For a longtime, the most prevalent explanation was related to the widely acceptable theory on aging, the Free Radical Theory. This theory attributes the aging process to the continuousaccumulation of oxidative damage in macromolecules generated by reactive oxygen species (ROS) produced in the mitochondria [179]. A later variation of this theory, the Oxidative Stress Theory, attributes the oxidative damage to the imbalance between preoxidant and antioxidant components, and CR was suggested to increase the resistance to oxidative stress [180]. Recently, this explanation was put into question, at least for rodents, as increasing oxidative stress by several genetic alterations increased aging-related diseases, such as cancer, but did not diminish life span [181-183]. ROS, primarily H_2_O_2_, have recently been suggested to promote aging as activators of the TOR (target of rapamycin) pathway [184]. This signaling pathway acts as a sensor of the nutritional and energetic state in the cell and transmits anabolic signals to regulate cell size, growth, and metabolism. Mammalian TOR (mTOR) could play an important role in the regulation of life span, as indicated by findings showing that CR attenuated mTOR signaling in several tissues in mice [185], and mice deficient of ribosomal protein S61 kinase 1, a central component in mTOR signaling, or mice treated with rapamycin, an inhibitor of the mTORC1 component, exhibited increased life span [186,187]. Interestingly, mTOR has also been recently linked to the circadian clock as a light-activated signaling cascade in the SCN of mice [188].

CR-fed animals resemble RF-treated animals, as they usually consume all or most of their food within a short period of time. While anticipating for food, calorically restricted animals show a rise in body temperature [189]. Thus, due to the temporal component of food intake, it is possible that CR, similarly to RF, synchronizes peripheral clocks and influences clock-controlled output systems, such as the anticipatory behavior and body temperature. As opposed to RF, CR entrains the clock in the SCN [190-192]. Under light-dark conditions and daytime feeding, calorically restricted mice showed strong FAA but with a phase advance of the nocturnal pattern of activity, a direct output of the SCN clock. When mice were transferred to dark-dark conditions, i.e. under free-running conditions, and fed *ad libitum*, the onset of the nocturnal period of locomotor activity occurred significantly earlier (1.3 h) in the calorically restricted than in *ad libitum*-fed animals, indicating an SCN effect. The period, however, did not differ between calorically restricted and *ad libitum*-fed mice [190]. Also, when SCN clock gene expression was tested, slight changes in gene expression were observed [191,192]. Overall, these results suggest that CR during the daytime affects the temporal organization of the SCN clockwork and circadian outputs in mice under light-dark cycle. In addition, CR affects photic responses of the circadian system, as measured by light pulses, suggesting that energy metabolism modulates gating of photic inputs in mammals [192]. It is noteworthy that microarray data comparing gene expression in seven different tissues under CR identified circadian rhythms among the three most over-expressed biological processes, with *Per2* being the most up-regulated gene [193]. Collectively, these findings suggest that synchronization of peripheral oscillators during CR could be achieved directly due to the temporal feeding, as has been reported for RF [143,152,153], or by synchronizing the SCN [190-192], which, in turn, sends humoral or neuronal signals to entrain the peripheral tissues [194,195]. It is not known whether there is dominancy or harmony between the central pacemaker and peripheral oscillators under CR.

## *C. Intermittent fasting (IF)*

During IF, also denoted alternate day fasting (ADF), food is available *ad libitum* every other day. IF-treated mice eat on the day they have access to food approximately twice as much as those having continuous access to food [196-198]. Similarly to calorically restricted animals, IF-fed animals exhibit increased life span in comparison with the *ad libitum-*fed control, even if there is little or no overall decrease in calories [199,200]. IF-fed animals also exhibit improved glucose metabolism, cardio-protection, neuro-protection [196,201-205], and increased resistance to cancer [197,200]. IF may also decrease the risk for cardiovascular diseases in humans [206].The IF-induced beneficial effects are thought to occur independently of the overall caloric intake, but the underlying mechanisms are still unknown. One suggested mechanism is stimulation of cellular stress pathways induced by the IF regimen [196,207,208]. Brain-derived neurotrophic factor (BDNF), normally involved in brain development and plasticity, is elevated in IF animals, and is causally linked to the protective effect of the IF regimen against neuronal damage inflicted by the neurotoxin kainic acid [209]. It must be noted, however, that BDNF could not be linked to the neuro-protective effects in the brain of calorically restricted rats [210,211], but increased levels of another neurotrophic factor, glial cell line-derived factor (GDNF), were correlated with neuro-protection of a calorically restricted primate model of Parkinson's disease [212]. Interestingly, BDNF is also a component of the hypothalamic melanocortin pathway that controls food intake and body weight in adult mice [213], and it has been implicated in the regulation of energy metabolism [214]. Heterozygousknockout BDNF (BDNF^+/-^) mice exhibit metabolic abnormalities, hyperphagia, obesity, and insulin resistance that could be significantly reversed by IF, indicating that BDNF is indeed involved in the beneficial effects induced by IF [214]. Interestingly**,**the BDNF^+/-^ mice resemble circadian *Clock* mutant mice [133] in metabolic abnormalities. In addition, BDNF and its cognate receptor TrkB were suggested to play a role in circadian modulation of the SCN pacemaker sensitivity to light [215,216]. These data point to the possibility that IF could affect the SCN and, as a result, peripheral clocks, at least via elevating BDNF levels.

Recently, we have shown that, under an IF protocol,when food was introduced during the light period, mice exhibited almost arrhythmicity in clock gene expression in the liver. Unlike daytime feeding, nighttime feeding yielded rhythms similar to those generated during *ad libitum* feeding [198] (Figure 3). Furthermore, rhythms were maintained when daytime IF occurred under disruptive light, suggesting that SCN signals were involved in inducing the arrhythmic state in the periphery [198]. Thus, the fact that IF can affect circadian rhythms differently depending on the timing of food availability and light conditions suggests that this regimen affects the SCN clock, similarly to CR. We assume that SCN resetting by IF and CR could be involved in the health benefits conferred by these regimens [195].

The effects of IF are in contrast to those of restricted feeding (RF) that dictates peripheral rhythms in arrhythmic and mutant mice and animals with lesioned SCN regardless of the lighting conditions [142,143,148-151] (Figure 3). It, thus, appears that IF is not as dominant as RF in dictating peripheral rhythms. Never-theless, this feeding regimen exhibits some similarities with RF, as reflected by the anticipatory feeding behavior that preceded food availability and restoration of circadian rhythms under disruptive light conditions, due most likely to the effect on the food entrainable oscillator (FEO) [198]. Thus, under daytime IF, clock gene expression in the periphery would be controlled by the SCN, which responds to both light-dark cycle and IF, as well as directly by the temporal feeding *via* the FEO. Co-activation of both the FEO and the SCN would yield rhythms at two opposite phases leading to overall arrhythmicity. In contrast, under nighttime IF, normal rhythms are generated, as both the FEO and the SCN work in synchrony to dictate peripheral rhythms [198].

## V. The circadian clock as a possible mediator in CR- or IF-induced increased longevity

## *A. Long-lived, spontaneously calorically restricted αMUPA mice*

 αMUPA mice carry as a transgene, the urokinase-type plasminogen activator (uPA) [217], an extracellular fibrinolytic serine protease implicated in tissue remodelling [218] and brain development and plasticity [219-224]. αMUPA mice spontaneously eat less (20-30%) compared to their wild type (WT) FVB/N control mice when fed *ad libitum,* indicating that their appetite is genetically suppressed. The mechanism linking transgenic uPA to reduced hunger is not yet clear. It could be related to uPA over-expression in the brain stem, as was found in two transgenic lines showing reduced food intake [225]. The transgenic effect is likely to be developmental, similarly to the remodelling effect recently detected in αMUPA developing incisor teeth [226]. αMUPA mice live longer (median, 16%; 10^th^ decile, 15%) than WT mice [227], thus resembling calorically restricted animals in showing an inverse relation betweenfood intake and life span.αMUPA mice exhibit additional similarities with calorically restricted mice, such as reduced body weight, reduced levels of serum IGF-1 or glucose, enhanced capacity to conduct apoptosis, and reduced incidence of tumors[225,227-230].

## *B. αMUPA mice, circadian rhythms, and aging*

**Figure 4.**
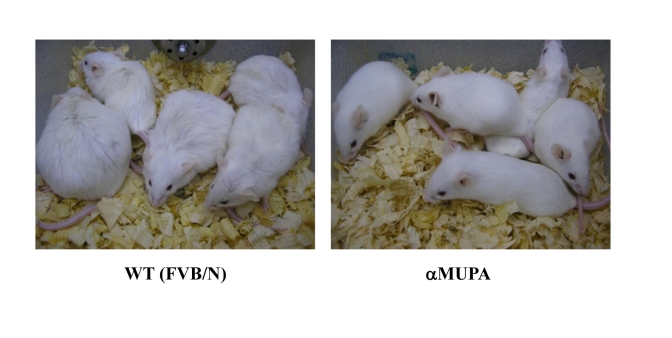
18-month-old αMUPA and FVB/N WT mice. αMUPA mice maintain a youthful and healthy appearance, whereas WT mice look old.

Recent data show that αMUPA mice exhibit higher amplitude in the circadian expression of several clock genes in the liver compared with FVB/N WT mice. This change coincides with higher amplitude rhythms of food intake and body temperature [194]. Since circadian patterns of food intake and body temperature constituteclock-controlled output systems, it is conceivable that their alteration in the transgenic mice stems from the higher amplitude of clock gene expression in the periphery, and possibly also in the central biological clock in the SCN. Higher amplitude of circadian rhythms have been previously associated with young age [15] and extended life span [19]. Support for a linkage between circadian rhythms and attenuation of aging in αMUPA mice is provided by comparing young vs. old mice. When tested for circadian food intake, an SCN output system, 18-month-old WT control mice exhibit a 4-6-h shift in circadian food intake compared to 5-month-old mice [195]. This behaviour is consistent with literature data showing that aging can alter the amplitude and/or phase of circadian rhythms [15,16,18]. In contrast to WT mice, adult and young αMUPA mice show similar circadian food intake, indicating that at least some aspects of circadian behavior maintain a youthful pattern at an old age in these mice. At an old age, αMUPA mice maintain a young and healthier appearance, they look lean, and their fur is shiny, whereas WT mice are sluggish and they look old (Figure 4). In addition, αMUPA mice do not become obese throughout their life-time, whereas about one third of the WT mice show severe obesity (Figure 4). The major difference in body weight between αMUPA and WT mice stems from the fact that the quantitative difference in food intake between αMUPA and WT mice is maintained at the old age.

## *C. αMUPA mice reveal effectsof feeding regimens on circadian rhythms*

**Figure 5.**
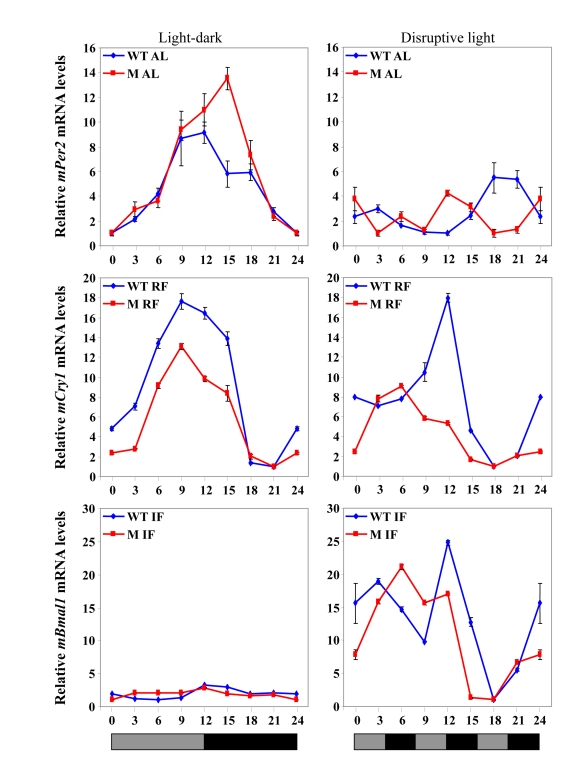
Clock gene expression in the liver under various feeding and lighting conditions in αMUPA (M) and WT mice. Expression levels of the following clock genes are presented under light-dark or disruptive light conditions:*mPer2*during *ad libitum* (AL) feeding, *mCry1* under RF, and *Bmal1* under IF.Total RNA extracted from liver tissue collected every 3 h around the circadian cycle (mean ± SEM; n=3 for each time-point and each mouse group) was reverse transcribed and analyzed by quantitative real time PCR. Clock gene levels were normalized using *Gapdh* as the reference gene.

It is difficult to eliminate the effect of temporal food consumption in calorically restricted animals, as mice consume their food within a few hours. αMUPA mice spontaneously consume reduced calories (20-30% reduction) compared with WT mice under different feeding regimens, i.e. AL, RF and IF, suggesting that these mice can be utilized as a model for CR in the absence of the imposed temporal food consumption under *ad libitum* feeding, and a model for imposed temporal CR under RF or IF conditions. Therefore, the transgenic mouse model αMUPA [225] has recently been used to investigate the contribution of calorie reduction *per se *vs. timed feeding to clock adaptation [49]. Under light-dark conditions and *ad libitum* feeding, αMUPA mice show high amplitude, appropriately reset circadian rhythms in peripheral clock genes [194] (Figure 5). This finding could reflect the effect of the reduced calorie intake on the SCN in αMUPA mice, as has been previously reported for calorically restricted animals [190-192].

Under light-dark conditions, RF advanced the expression phase of all clock genes in the liver in a similar manner in both WT mice and αMUPA mice, and in some cases increased the amplitude [49](Figure 5). These findings were in concert with previous results in the literature, as is mentioned above. Nevertheless, an effect of the SCN on clock-controlled output systems under RF could be seen when the phases of peripheral clock gene expression in WT mice vs. αMUPA mice were compared under arrhythmicity imposed by disruptive light. Whereas the pattern of clock gene expression did not change in WT mice, a phase shift was seen in several genes in αMUPA mice (Figure 5), suggesting an SCN effect. Again, this effect of the SCN could be attributed to the reduced calorie intake of αMUPA mice. Altogether, these findings suggest that both the reduced calories and the light-dark cycle work in synchrony on the central biological clock of αMUPA mice to generate rhythms in the periphery. However, it seems that in WT mice, as has been found for other mouse strains, RF is dominant over the SCN in dictating rhythms in the periphery regardless of the lighting conditions. In αMUPA mice, RF dictates the phase of clock gene expression under light-dark; but under disruptive light conditions, as the SCN is under the influence of calorie restriction, it becomes dominant in dictating the phase of clock gene expression.

Unlike RF, the effect of IF on circadian rhythms in αMUPA mice was similar to that of WT mice under light-dark or disruptive light conditions, and it resembled also that on C57BL mice (Figure 3, Figure 5). Thus, in all mice tested, daytime IF caused arrhythmia in clock gene expression in the liver under light-dark, whereas rhythmicity was restored under disruptive light [198] (Figure 3). These observations suggest that IF, similarly to CR, may affect the SCN clock. This effect could possibly be mediated through a metabolic state generated by the day of fast during IF regardless of the calories consumed, as discussed earlier.

Altogether, the findings in αMUPA mice suggest that reduced calories affect the SCN so it becomes dominant over RF in the periphery only under disruptive light conditions. In addition, IF affects peripheral rhythms depending on the timing of food availability and light conditions, but regardless of the total daily calorie consumption, suggesting that this regimen induces a metabolic state that affects the SCN.

## D. Temporal vs. quantitative food consumption and circadian rhythms

Previous publications have dealt with the issue of timed feeding and life span, reporting that calorically restricted mice showed increased longevity whether fed twice a day at daytime, once a day at daytime or nighttime, several times a day at nighttime [231,232], or three meals a week [233]. In these studies, the low-calorie feeding was practically timed and confined to the day or night, similarly to MUPA, or introduced in large intervals and continued throughout life time allowing appropriate adaptation. As a result, timed feeding was suggested to lead to high amplitude circadian rhythms and increased life span [232,234]. However, others rejected any contribution of timed feeding to CR-induced longevity [174,231]. The uncoupling of timed meals from reduced calories could practically be achieved only with animals, such as αMUPA, that spontaneously eat less. The results obtained with αMUPA indicate that temporal and quantitative aspects of food intake can be separately controlled. The timing of food intake is controlled by the central biological clock, whereas a separate mechanism appears to dictate the amount of food or calorie intake, that, in turn, could entrain the SCN clock, as has experimentally been shown for calorically restricted animals [191,192]. The results achieved with IF suggest that IF can be beneficial when food is given during the activity period of the animal, as explained above. Indeed, neuro- and cardio-protection alongside increased fatty acid oxidation and improved stress resistance have been induced after weeks of IF treatment when food was introduced at the beginning of the activity period [205,235-237]. It is noteworthy that cardio- and neuro-protection and life span extension were also seen when food was introduced during the day, but after many months of IF treatment [196,199], so that the animals could adjust after such a prolonged treatment. In light of these findings, we assume that the effect of IF on the SCN through a metabolic change, as mentioned above, alongside the timed feeding might affect the SCN to yield better-reset rhythms.

## E. Differences between αMUPA mice and calorically restricted rodents

Although αMUPA mice exhibit reduced calorie intake and body fat, they show remarkable differences in energy metabolism compared with CR-treated animals. In particular, calorically restricted animals exhibit high levels of ghrelin [238,239], but low levels of leptin [240,241]and insulin [173], indicating an overall state of hunger. It is noteworthy that leptin-deficient animals are long-lived under CR feeding regimen, suggesting that leptin is not necessary for the CR-mediated benefits [242,243]. In addition, CR-treated mice exhibit high expression levels of PGC-1α and no change in PPARγ levels in the liver [244,245]. All these findings are in sharp contrast with those found in αMUPA mice that have low levels of ghrelin and high levels of leptin and insulin, suggesting that αMUPA mice eat less because their metabolism is of satiated rather than hungry animals (unpublished data). Nevertheless, one aspect found to be common to both αMUPA mice and calorically restricted animals are the low SIRT1 expression levels in the liver [246] and high levels in the brain [247]. It is noteworthy that the information regarding SIRT1 levels in the hypothalamus of calorically restricted animals is still lacking, and data for peripheral tissues is sometimes contradictory [124,246]. Results obtained with SIRT1-null micehave recently suggested that this enzyme could be required for the *in vivo* response to CR [248], and transgenic mice over-expressing SIRT1 show a phenotype resembling calorically restricted animals [249]. We assume that, in calorically restricted mice, SIRT1 activity could be elevated in the brain, possibly in the hypothalamus and SCN, through AMPK activation, as AMPK can be activated in the hypothalamus under starvation conditions and high ghrelin [112,121]. In αMUPA mice, that show low AMPK levels in the hypothalamus, the high leptin levels could lead to SIRT1 elevation, as leptin is required for the increase in SIRT1 protein levels in the hypothalamus under starvation [250]. Thus, although stimulated through different pathways in αMUPA and calorically restricted mice, SIRT1 could act as a common factor modulating the SCN clock and, as a result, longevity.

αMUPA mice share also some similarities with those of Lou/C rats, both obesity-resistant long-lived rodents.

**Figure 6.**
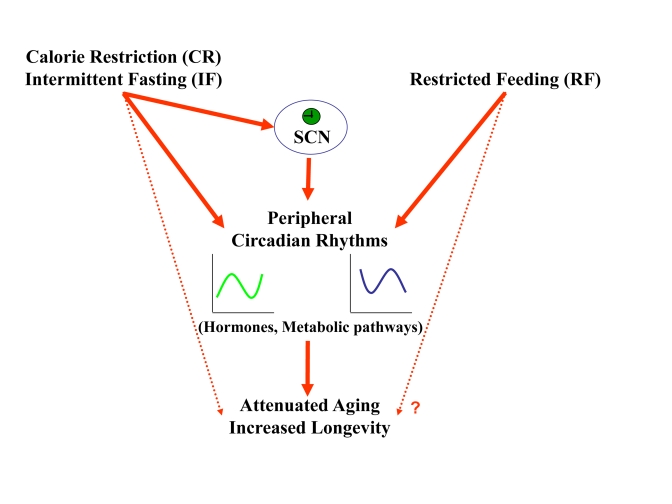
A schematic model describing the effect of feeding regimens on longevity through peripheral and SCN clock resetting. CR and IF reset circadian rhythms in the periphery and the SCN. The synchronized, robust circadian rhythms could be the mediator though which these feeding regimens lead to aging attenuation and life span extension. RF resets circadian rhythms only in the periphery, but its effect on life span is not known.

However, there are some fundamental differences in Lou/C rats compared with αMUPA mice, such as increased levels of PGC-1α and SIRT1 in the liver, increased levels of ghrelin, and reduced levels of leptin and insulin in the serum, although with some improved sensitivity for the latter two hormones [251,252].Overall, it seems that αMUPA mice are metabolically different from Lou/C rats.

## F. Role of circadian rhythms in CR on health and longevity

 The capacity of CR to reset the SCN clock, as previously reported [191,192],and the pronounced circadian rhythms seen in the long-lived αMUPA mice, pose the biological clock as a possible major factor determining longevity of calorically restricted mice [194,195]. The beneficial effect induced by CR on health and longevity can be achieved by appropriately resetting and synchronising a variety of hormonal, biochemical, and physiological functions. In turn, some of these functions can feedback to the biological clock in the periphery [143,153] and the SCN [191,192] and help sustain the rhythms. Indeed, the redox state affects the dimerization of the two clock proteins CLOCK and BMAL1 *in vitro *[96]. As SIRT1 has been linked to life span and suggested to mediate CR-induced effects [115,126,253], it could be a candidate that modulates the clock of calorically restricted animals, as discussed above. Clock resetting could lead to robust circadian rhythms that are associated with young ages and extended life span [15,16,19,195].

## VI. Conclusions

RF entrains peripheral clocks due to temporal food consumption, whereas CR and IF appear to synchronize the central pacemaker in the SCN, suggesting a role for a metabolicstate imposed by low calories in central clock entrainment. In αMUPA mice, reduced calories alone were found not to be sufficient to sustain rhythms, unless feeding was spontaneously timed at night, or timed at day through a restricted feeding protocol. Therefore, it appears that when reduced calories are timed, as always occurs during CR and IF regimens, clock adjustment can influence a wide variety of output systems, so that cellular and physiological systems perform in a more synchronised and appropriately reset manner. We assume that SIRT1 could be a key mediator in clock synchronization at least under CR. Robust circadian rhythms can ensure a better tissue and body homeostasis, and could constitute an important mediator in aging attenuation and longevity extension (Figure 6).
